# Les Misérables: An analysis of low SWB across the world

**DOI:** 10.3389/fpsyg.2023.1107939

**Published:** 2023-06-08

**Authors:** Georgios Melios, Kate Laffan, Laura Kudrna, Paul Dolan

**Affiliations:** ^1^Department of Psychological and Behavioural Science, London School of Economics and Political Science, London, United Kingdom; ^2^The Gallup Organization, Charlottesville, VA, United States; ^3^Institute of Applied Health Research, Birmingham University, Birmingham, United Kingdom

**Keywords:** subjective wellbeing, low life satisfaction, misery, hierarchical models, Gallup World Poll

## Abstract

Global trends indicate that the prevalence of low subjective wellbeing is on the rise, though not all regions are equal in terms of both absolute levels and their trajectories. In this paper, we explore the relative importance of individual- and country-level factors in predicting low SWB. Put differently, we ask if a person found themselves behind a veil of ignorance, should they want to know who they will be or what country they will live in to better understand their risk of having low wellbeing. To answer this question, we leverage data from the most extensive wellbeing survey in the world—the Gallup World Poll. We explore people's likelihood of reporting low evaluative wellbeing (that their life is close to the worst possible life on the Cantril ladder) and low experiential wellbeing (reporting having felt angry, sad, stressed, and worried for most of the day yesterday). Using multilevel models on both measures, we show that individual factors have the greatest explanatory power across both measures, but that country level factors are almost four times more important in explaining the variation in low evaluative wellbeing than low experiential wellbeing around the world. We also present evidence that individual and country-level factors interact, suggesting that a complex system of people and places determines people's likelihood of reporting low SWB.

## 1. Introduction

The science of “happiness” or subjective well-being (SWB) was born, in part, out of a rejection of psychology's historical focus on mental ill-being, as well as of the use of economic indicators as the sole measures of societal progress (Veenhoven, 1996). This science has contributed much to our understanding of what constitutes the “good life” or, in other words, what makes happy people happy (Diener et al., 2018b), and SWB indicators are increasingly widely considered social indicators of primary importance (Boarini et al., 2012; Stone and Mackie, 2013). Given the inroads that have been made, we argue that it is time to employ the data and approaches that have contributed to this science to understand who is at risk of missing out on the good life altogether. The low wellbeing of these people and its determinants have been largely unexplored by the SWB literature to date, despite some existing evidence showing that the drivers of SWB vary across the wellbeing distribution (Dolan et al., 2008; Binder and Coad, 2011). Our argument is well-aligned with the increasing emphasis being placed on the negative quality of life indices and trends in the broader social indicators movement (Glatzer et al., 2015; Land and Michalos, 2018), for example, in the work of Anderson (2015) which examines the poor quality of life in terms of both low SWB and other objective indicators. Those suffering from low wellbeing are also of policy importance: identifying those who fall within this group can inform efforts to address disadvantage (Dolan et al., 2022).

SWB measures include both people's evaluations of their lives and reports of their experiences as they go about them (Dolan et al., 2017). People with low SWB can, therefore, be identified as those who evaluate their life poorly and/ or report negative experiential wellbeing on a day-to-day basis. Longitudinal data from the World Happiness Report indicate that on both counts, low SWB is on the rise around the world, though these trends vary across regions (Helliwell et al., 2019). These trends are ascertained from data from the Gallup World Poll (GWP)—an annual survey conducted from 2005 to 2021 that is, as a result of a probabilistic sampling strategy, representative of 98% of the world's population. The sample includes 164 nations, with a sample size of more than 1.8 million observations. It is the largest and most representative sample of wellbeing data from the world's population available. The poll includes responses to the Cantril ladder question, which asks people to rate their life on a ladder, the bottom rung representing the worst possible life and the top the best. The poll also captures individuals' reports of both positive and negative experienced wellbeing yesterday.

The GWP data provide an unparalleled resource with which to examine and better understand the SWB of the world. They have been productively used to investigate the role of specific determinants of wellbeing around the world, including marriage, employment, prosociality, and life meaning (Jebb et al., 2020), and separately, health (Joshanloo and Jovanović, 2021), age (Blanchflower and Graham, 2020), food insecurity (Frongillo et al., 2017), social engagement and air pollution (Xia et al., 2022), and inequality (Gluzmann and Gasparini, 2018), among other factors. They have also been used to track trends in wellbeing across the life-course (Deaton, 2018) and in how people are faring globally (Helliwell et al., 2019). Almost no work, however, has leveraged the Gallup World Poll to investigate the risk and protective factors, at both the individual and country level, for falling among the worst off around the world.^1^

The literature leaves many questions outstanding regarding what puts people at risk of low SWB, but a first-order question is the relative importance of individual and country-level factors. Put differently, if you were in Rawls' position behind a veil of ignorance, should you want to know who you will be or what country you will live in to better understand your risk of falling into that category? (Rawls, 2020). Though existing work with Gallup has examined individual and country-level factors (e.g., Deaton, 2008), we are unaware of any work to date which has directly examined a range of possible risk and protective factors to compare their relative power at predicting low SWB.

Many of the strongest predictors of average SWB that have been identified by the literature to date are individual-level factors. For example, whether someone is healthy, socially connected and employed have all been highlighted as key determinants of SWB (Dolan et al., 2008, 2022). People living in urban compared to rural areas typically report lower SWB, as do men compared to women (Joshanloo and Jovanović, 2020; Okulicz-Kozaryn and Valente, 2021). Income has also been shown to have a association with SWB, particularly at the bottom end of the income distribution, with most prior studies finding a log-linear relationship (Stevenson and Wolfers, 2013), and some studies suggesting that the marginal benefits of income decrease with income (Jebb et al., 2018). Additionally, much of the health and economic literature on SWB provides evidence of the popular notion of the midlife crisis, with both younger and older adults having better SWB than those in their 40s and 50s (López Ulloa et al., 2013). Finally, research indicates that a substantial proportion of the variance in SWB can be explained by genetic factors (De Neve et al., 2012).

By comparison, the associations between country-level factors and SWB are typically smaller in magnitude. For example, in their study of country-level air pollution using the Gallup World Poll, Xia et al. (2022) report standardized individuals' health on happiness of 0.094, compared to 0.050 from country level air quality. The same study also reports standardized coefficients of 0.037 of income, compared to 0.027 of GDP at the country level. Why might this be the case? People may be more prone to adapting to country-level factors—adjusting to both the positive and negative features of their country—than they are to their own life circumstances (Headey, 2010). Although existing work does identify substantial adaptation to some individual life events including bereavement and divorce too, not others such as becoming unemployed (Luhmann et al., 2012). Adaptation can be understood as a process of withdrawal of attention *via* explanation (Dolan et al., 2021). Except in extreme circumstances such as war or famine (Matanov et al., 2013; Shemyakina and Plagnol, 2013), many country-level factors may not be as regularly attention-seeking as individuals' circumstances, like health, and might be easier to explain away given that people have less agency over country-level factors than their own circumstances, therefore potentially undermining their relative importance for predicting low SWB.

Research into the determinants of wellbeing across evaluative and experiential measures indicates that the relative importance of life circumstances including, for example, income, health, and employment status, varies substantially across these different dimensions of wellbeing (Dolan et al., 2017; Miret et al., 2017; Macchia et al., 2020). These findings emphasis the importance of adopting a multidimensional approach to modeling SWB, irrespective of whether the focus is on average or low SWB. Existing work which compares the country-level determinants of average evaluative and experiential wellbeing measures using the GWP finds that external factors like governance and community context are more closely related to evaluative wellbeing (Diego-Rosell et al., 2018). In other words, these factors are relatively more important when people reflect on their lives than when they report the emotions they experienced on the previous day. A potential explanation for this is that evaluative measures of SWB, and in particular, the Cantril ladder question contained in the GWP, involve greater levels of social comparison.

Based on the existing literature, our first hypothesis is that more of the variation in low SWB will be explained by individual-level factors than by country-level ones (H1a). Many country-level factors like politics and the state of the economy are likely to be more closely related to evaluative than experiential well-being, on account of their being more salient when making cognitive evaluations of one's life as compared to how often they come to mind over the course of a day (Stone and Mackie, 2013). As a result, we further hypothesize (H1B) that country-level factors will have greater predictive power in relation to low evaluative, compared to experiential, wellbeing. To examine these questions we estimate null three-level logistic models which account for the fact that responses are clustered in both countries and years. The null or empty model contains just one fixed term—the mean—and then a variance component at each of the 3 levels (individuals, countries, years). This allows us to calculate the proportion of the total variation explained by the country level, the year and also how similar individuals within a country and a year are on the two outcomes. In order to further examine the relative importance of individual, country-level and year factors, we also carry out the same analysis broken down by region.

H1a: The variation in low SWB will be better explained by individual-level factors than country-level ones.
H1b: The variation in low evaluative wellbeing will be explained by country-level factors to a greater extent than the variation in low experiential wellbeing.

This approach decomposes unexplained variation, but as highlighted above the SWB literature offers insights into the key determinants of average SWB. These insights are likely informative about the correlates of low SWB too. We go on to incorporate several of the individual factors that are commonly examined in the SWB literature: income, gender, age, health issues, support, marital status, retirement status, and whether they live in an urban area. We examine the explanatory power of these determinants both on their own and when we include Gross Domestic Product (GDP) per capita at the country level. GDP is the most widely used conventional economic measure of social progress and has been the focus of a large body of literature that aims to understand differences in average well-being around the world (Stiglitz et al., 2009; Deaton and Stone, 2013; Stone and Mackie, 2013). By exploring the extent of the unexplained variation in our original models that GDP accounts for once we include it we are able to comment on its relative importance across both measures of low SWB.

Finally, the SWB literature indicates that individual, time and country-level factors may interact such that certain individual factors matter more or less depending on the country you live in or the period under consideration. Existing research suggests that the relationship between who people are (demographic characteristics like age or gender) and their SWB depends upon their social and economic contexts. For example, contrary to much of the evidence on the U-shape pattern between age and SWB, Steptoe et al. (2015) find that people living in former Soviet Union countries and eastern Europe have worse SWB with age—but in Latin America, there was little difference in SWB across the life-course. Gender differences in SWB have been also shown to depend on whether people live in a place with strong gender rights (Graham and Chattopadhyay, 2013). The relationship between unemployment and SWB has also been shown to vary across time according to economic conditions of the period (Arrondo et al., 2021).

In this work, we add to this literature by focusing on the role of income. Income is not the only individual-level factor that we would expect to vary in importance across countries but given that factors such as welfare provision and the cultural importance placed on income and wealth vary substantially across countries and regions (Duffy and Gottfried, 2013; Dollar et al., 2015), it is interesting to consider whether the relationship between how much people earn and their probability of reporting low SWB varies across countries too.

Our second main hypothesis is that income is differently related to low SWB in different countries around the world. To examine this we run a random slope model that allows for cross-country differences in the relationship between income and both measures of low SWB. This approach allows us to investigate how what people earn and the country that they live interact to protect or enhance the risk of people experiencing low SWB.

H2: The relationship between individual income and low SWB varies across countries.

Taken together, these investigations help us better understand the geography of low subjective well-being around the world, while also shedding light on differences across evaluative and experiential dimensions of well-being in different regions. In what follows, we present the data in Section 2, analysis in Section 3 and discuss the results in Section 4.

## 2. Data

To explore cross-country differences in low SWB, we use data from GWP between 2005 and 2021. Countries were sampled to represent the population of each nation. The sample includes 164 nations, with a total sample size of more than 1.8 million observations, the largest and most representative sample of the world available.^2^ We draw on relevant survey variables from the GWP that were polled across most nations. Table 1 includes a variable list and respective descriptive statistics.^3^

**Table 1:** Summary statistics.

**Statistic**	***N***	**Mean**	**St. Dev**.	**Min**	**Max**
Low eval. SWB	1,835,719	0.289	0.453	0	1
Low exp. SWB	1,835,719	0.071	0.257	0	1
Household size	1,835,719	4.137	2.681	1	22
Income (quintiles)	1,817,483	3.229	1.418	1	5
Gender (Female = 1)	1,835,719	0.533	0.499	0	1
Age	1,835,719	41.409	17.604	15	99
Subjective health	1,835,719	0.246	0.431	0	1
Count on help	1,800,231	0.831	0.447	0	4
Worry	1,835,719	0.379	0.485	0	1
Sadness	1,835,719	0.234	0.423	0	1
Stress	1,835,719	0.331	0.470	0	1
Anger	1,835,719	0.199	0.399	0	1
Retired	1,835,719	0.199	0.399	0	1
GDP (log)	1,835,719	8.685	1.400	5.641	11.595

### 2.1. Measures

The GWP identifies individuals as suffering if they report that their current and future (in 5 years) satisfaction with life is 0–4 in the Cantril ladder (0–10). For this research, we define low SWB based on if they report a 0–4 life satisfaction only currently and not in the future to focus on current states of wellbeing. We recode this measure into a binary variable of 0 (not low) and 1 (low). In the Supplementary Table 7, we test whether using the GWP's measures of suffering or a threshold of 0–3 for low evaluative wellbeing alters any of our analyses and results remain substantively the same.

In addition to this definition, our paper explores cross-country differences in low experiential wellbeing. We define this measure based on individuals that report they experienced yesterday all of the negative emotions asked in the GWP: sadness, worry, stress, and anger. Using questions about feelings yesterday is the standard approach to capturing experiential SWB in large surveys. Inquiring into yesterday lessens the impact of survey effects on reports of experiential wellbeing, while still capturing feelings close to the time frame of interest thus mitigating recall bias (Stone and Mackie, 2013). We create a new binary variable that indicates that they have low experiential wellbeing (1) if an individual reported yes in all 4, otherwise not (0). In the Supplementary Table 7, we test whether using an alternative threshold of reporting at least 3 out of 4 of the negative emotions yesterday substantively influences our results. It does not.

**Figure 1:**
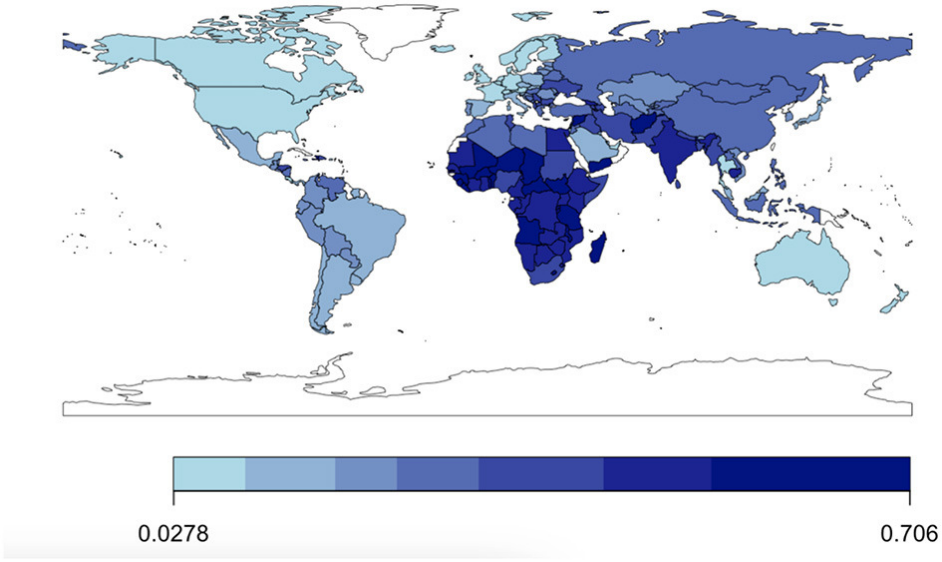
Low evaluative SWB across the world (mean values for 2005–2021).

**Figure 2:**
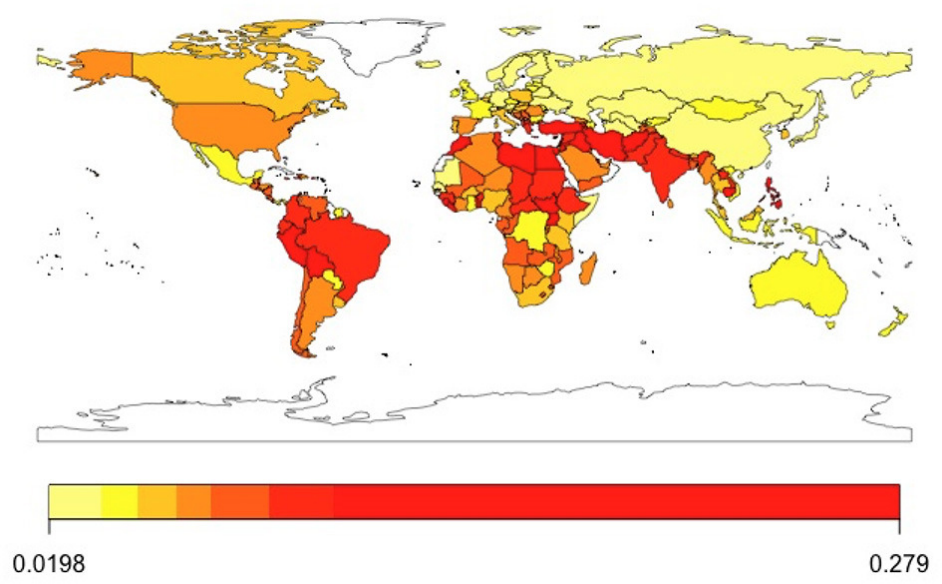
Low experiential SWB across the world (mean values for 2005–2021).

Summary statistics suggest that on average about 28.9% of the world population surveyed through the GWP evaluates their life poorly. In comparison, only about 7.1% of respondents experience it poorly as measured by whether they experienced anger, sadness, worry, and stress yesterday. Looking at Figures 1, 2, we observe great disparities around the world in the levels of low evaluative and experiential wellbeing. Supplementary Figures 7–9 show the evolution of low SWB by year as well as by year per global region. In addition to that Supplementary Figures 10–14, show the evolution of that variation over time by country in different global regions, respectively.

## 3. Analysis

In psychology, economics, and other social sciences, we often observe data with natural groupings, nested data (i.e., students nested in different schools, firms nested in states, individuals nested in different countries). Accounting for this data structure is important on both theoretical and statistical grounds. Ordinary Least Squares (OLS) regression models assume the independence of units, an assumption that is often violated when we have such structures. For example, individuals living in the same country probably have similar outcomes (or more similar than a random sample). These similarities could be due to shared institutional frameworks, economic conditions, language, culture, education, history, and others. Since this violates the independence assumption of OLS, ignoring the nesting might lead to biased estimates. A way to account for the bias is multilevel modeling (Mehmetoglu and Jakobsen, 2016).

In addition, this hierarchical structure of the data can actually help us understand important insights about the population at hand as it allows for a clear decomposition of variation. Knowing how much variation is due to each level can inform both theory and policy. In this case, we use multilevel models to understand how important living in a country is in explaining people's probability of low SWB.

Imagine on one extreme, you observe a country where virtually everyone has low wellbeing and another where the opposite is true. This could imply that country of residence determines their low SWB or lack thereof. At the other extreme, all of the variation could come from the individual, implying that countries have no role to play in people's probability of reporting low SWB. Using a multilevel model that accounts for the nested nature of the data at the country level and years across our sample allows us to estimate these different sources of variation.

To explore the variance across different levels in a multilevel model, we compute the variance partition coefficient (or inter-class correlation coefficient—ICC). ICC represents the proportion of the total variability in the outcome that is attributable to each level. In other words, the country ICC indicates how strongly residents of the same country resemble one another in terms of their low SWB. Therefore, the higher the country-level ICC the more similar citizens within countries are.

In a linear model, the ICC is based on the clear distinction that exists between the individual-level variance and the country-level variance (Rasbash et al., 2003; Merlo et al., 2006). In our case, our low SWB measures are coded as binary variables. In multilevel linear regression, the individual and area-level variances are expressed on the same scale. Therefore, the partition of variance between different levels is easy to perform for detecting contextual phenomena. In multilevel logistic regression, however, the individual level variance and the area level variance are not directly comparable. Whereas, the area level residual variance *V*_*A*_ is on the logistic scale, the individual level residual variance *V*_*I*_ is on the probability scale. Moreover, based on Merlo et al. (2006), *V*_*I*_ is equal to π (1–π) and therefore depends on the prevalence of the outcome (probability).

We follow the linear threshold model method as proposed in Goldstein et al. (2002) and expanded in Merlo et al. (2006) in order to be able to identify the ICCs. This involves converting the individual-level variance from the probability scale to the logistic scale, on which the area-level variance is expressed. In our case, the method assumes that the propensity for low SWB is a continuous latent variable underlying our binary response. In other words, every person has a certain propensity for low SWB, but only persons whose propensity crosses a certain threshold actually report it. ICC equals the proportion of well-being variance explained by unobserved individual variables. The unobserved individual variable follows a logistic distribution with individual-level variance *V*_*I*_ equal to π2/3 (that is, 3.29) (Merlo et al., 2006). On this basis, the ICC is calculated as:


(1) $$\begin{array}{l} ICC=\frac{V_{A}}{V_{A}+3.29} \end{array}$$

### 3.1. Cross country variations

Using this approach, we compute the ICC in our dataset for an empty three-level model (individuals nested in countries, nested in years) for low SWB using Gallup's survey weights to minimize bias. Table 2 reports the results. The ICC for the two measures provides a very interesting insight. Where individuals live (that is, country-specific level variance) explains almost 25% of the variance in low evaluative wellbeing (measured as low life satisfaction today, 0–4 in the Cantril ladder) but only about 9% of the variation in low experiential wellbeing (measured as the combined experience of anger, stress, sadness, and worry yesterday). Across both metrics, time only explains a small fraction of the overall variance.

**Table 2:** Understanding variance decomposition by level.

	**Individuals**	**Country**	**Years**
**Evaluative SWB**			
Variance	3.29	1.03	0.01
ICC	75.92%	23.87%	0.21%
Observations	1,835,719		
**Experiential SWB**			
Variance	3.29	0.31	0.01
ICC	90.47%	8.56%	0.97%
Observations	1,835,719		

### 3.2. Cross regional variations

**Figure 3:**
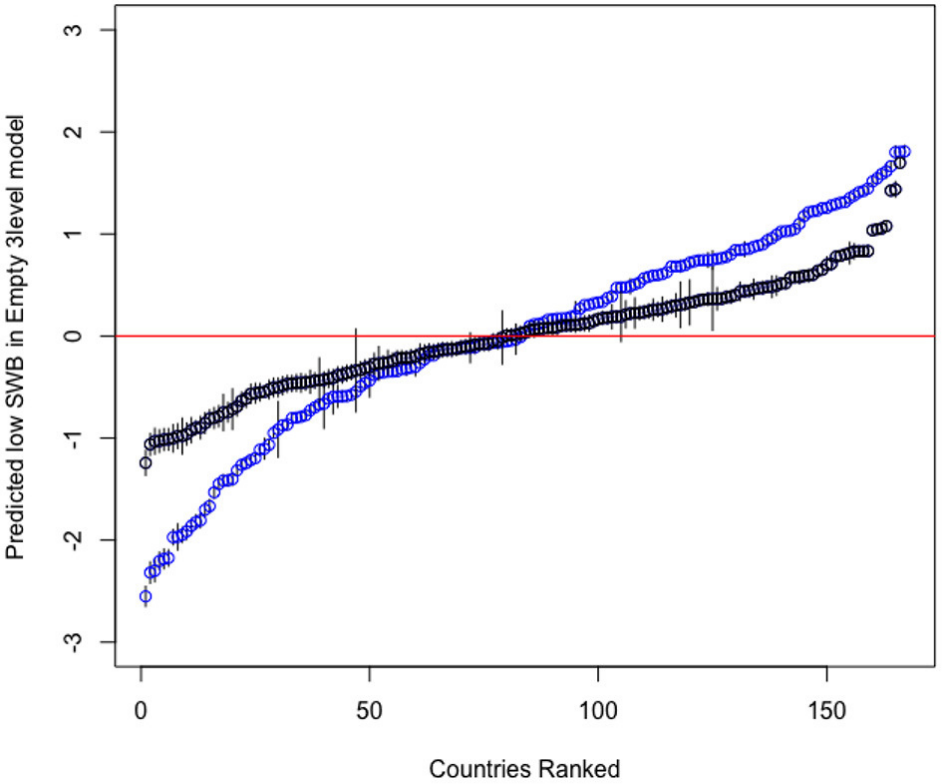
Predicted low SWB across the world.

Table 3 and Figure 3 suggest that the predictive power of country characteristics varies across different regions of the world and individual countries. Results of the empty random models suggest that country characteristics vary substantively in predicting evaluative vs. experiential wellbeing across different world regions. In industrialized economies such as in European countries and North America, country characteristics can explain about 1/5th of the variance in low evaluative wellbeing, whilst in sub-Saharan Africa, the predictive power of country characteristics can only explain <5% of the variance. With respect to low experiential wellbeing, the variance explained by country characteristics across different regions of the world remains below 8% with the exception of sub-Saharan Africa.

**Table 3:** Understanding variance decomposition by global region.

	**Individuals**	**Country**	**Years**
**Europe and North America**			
Evaluative SWB	77.51%	20.60%	1.89%
Experiential SWB	91.69%	7.80%	0.51%
**Asia & Indep. Com. States**			
Evaluative SWB	85.05%	14.93%	0.01%
Experiential SWB	90.76%	6.61%	2.63%
**Middle East and North Africa**			
Evaluative SWB	81.32%	18.19%	0.49%
Experiential SWB	92.14%	6.74%	1.12%
**Sub-Saharan Africa**			
Evaluative SWB	93.53%	5.57%	0.9%
Experiential SWB	84.70%	10.30%	5.10%
**Latin America**			
Evaluative SWB	90.51%	9.30 %	0.19%
Experiential SWB	94.92%	4.30%	0.77%

There are many differences between the regions of the world under consideration, including dimensions like institutional capacity, climate, and economic stability. It is beyond the scope of the current work to examine the potential explanatory factors behind the differences in the explanatory power of individual and country-level factors in these places. Recent evidence (Berggren and Bjørnskov, 2020) shows, however, that institutional quality and state capacity significantly affect SWB. Based on data from the Quality of Governance database, institutional quality in sub-Saharan Africa is in almost all countries^4^ homogeneously low (26,66 percentile rank on average in 2021). In addition to that, an important point of difference between the regions in which our findings diverge to the greatest extent, i.e., Sub-Saharan Africa and Europe and North America is the underlying prevalence of low evaluative and experiential wellbeing. The majority of people report low lie evaluations in Sub-Saharan Africa compared to just 14% in Europe and North America. Less stark but still substantive differences exist across these regions in experiential wellbeing: 7.2% of Sub-Saharan Africa reports low experiential wellbeing while 4.5% do in Europe and North America 4.5. Statistically speaking, the greater prevalence of both low life evaluations and low experiential wellbeing in Sub-Saharan Africa might partly explain why in low institutional quality states, where the scope and role of government in increasing SWB is limited, country-level factors explain less of the variation in low SWB.

Both the mean and by region variance decomposition suggest high heterogeneity between countries. To examine this heterogeneity, we use the empty models to obtain the predicted values of low SWB by country and plotted them against the respective intercepts of the empty models (ranked by predictive power). Figure 3 shows the results confirming the relatively higher importance of country characteristics in explaining evaluative in comparison to experiential wellbeing. Figure 3 also suggests that this difference in predictive power arises from the fact that at both tails of the predictions' distribution, country characteristics can predict either much higher (or lower) levels of low evaluative wellbeing than of experiential wellbeing.

### 3.3. Individual level predictors

To explore the effect of individual and country-level explanatory variables on low SWB, we now expand our model and introduce explanatory variables on income (measured according to which country-level income quintile the person falls into), gender, age, health issues, social support, whether the person is retired, married and lives in an urban area, as well as per capita GDP in their country measured 2015 US dollars. Table 4 shows the results using cluster-robust SE following (Huang and Li, 2022). Including solely individual-level controls for predictors of low SWB suggests a very similar variance decomposition as the empty models. However, including per capita GDP in log^5^ as a country predictor, the percentage of variance that explains low evaluative wellbeing attributed to countries decreases to 8%.

**Table 4:** Probability of reporting low SWB.

	**Dependent variable**			
	**Eval. SWB**	**Exp. SWB**	**Eval. SWB**	**Exp. SWB**
Income (in quintiles)	−0.254^***^	−0.164^***^	−0.257^***^	−0.164^***^
Gender (Female = 1)	−0.130^***^	0.260^***^	−0.138^***^	0.286^***^
Age	0.010^***^	0.046^***^	0.028^***^	−0.002^***^
*Age* ^2^	0.010^***^	0.046^***^	0.028^***^	−0.002^***^
Health issues	0.428^***^	0.821^***^	0.430^***^	0.819^***^
Count on help	−0.548^***^	−0.551^***^	−0.539^***^	−0.568^***^
Retired	−0.050^***^	−0.152^***^	−0.009^**^	−0.247^***^
Married	−0.203^***^	−0.148^***^	−0.247^***^	−0.045^***^
Urban	−0.185^***^	0.091^***^	−0.183^***^	0.099^***^
GDP per cap. in 2015 $ (log)			−0.740^***^	−0.152^***^
Constant	0.075	−3.204^***^	6.085^***^	−1.038^***^
Observations	1,635,201	1,635,201	1,622,269	1,622,269
ICC (country)	25.17%	8.03%	7.98%	7.52%
ICC (year)	0.01%	0.99%	0.08%	0.01%
Log likelihood	−814,960	−379,413	−807,083	−376,056
Akaike inf. crit.	1,629,942	758,850	1,614,192	752,136
Bayesian inf. crit.	1,630,078	758,998	1,614,352	752,283

The table shows the results for different choices of controls. Standard errors are in parentheses.

^*^p < 0.05;

** *p* < 0.01;

*** *p* < 0.001.

### 3.4. Income heterogeneity

**Figure 4:**
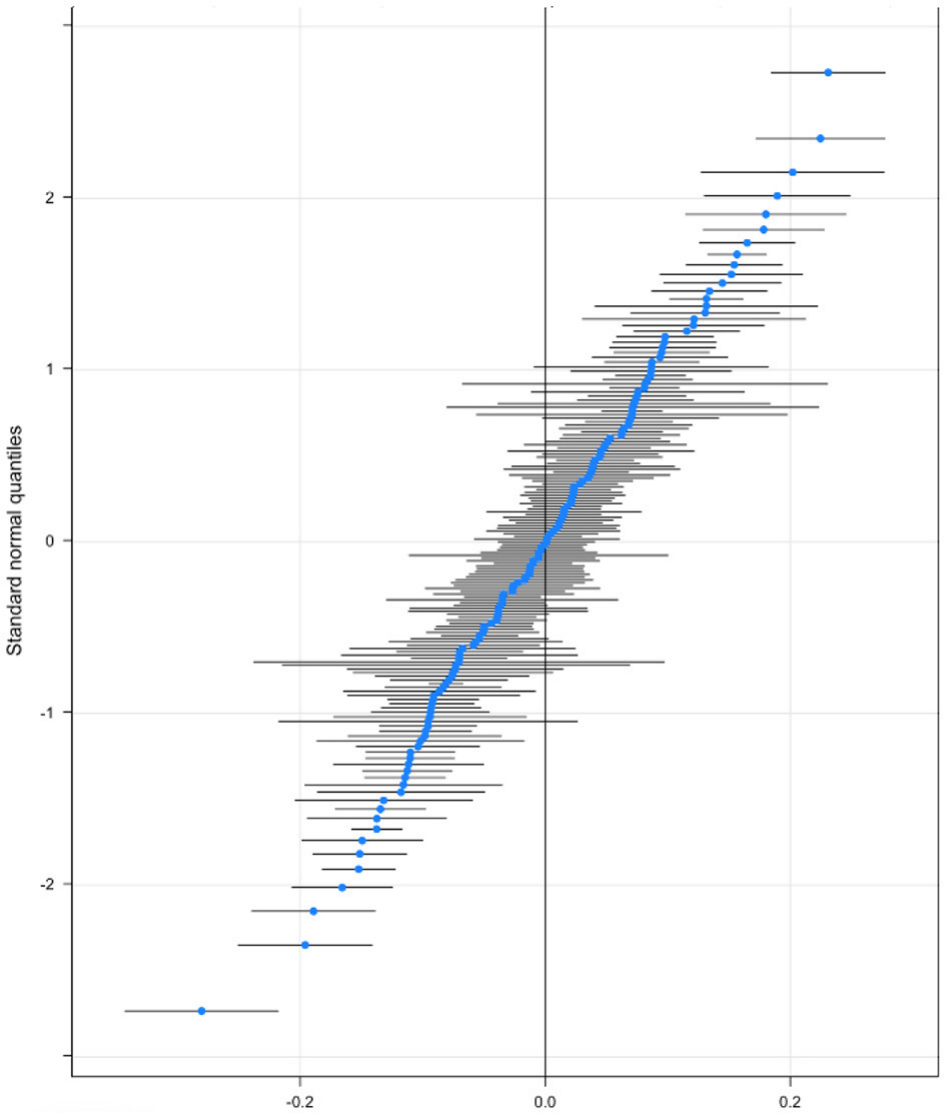
Random slope of income on low evaluative SWB.

**Figure 5:**
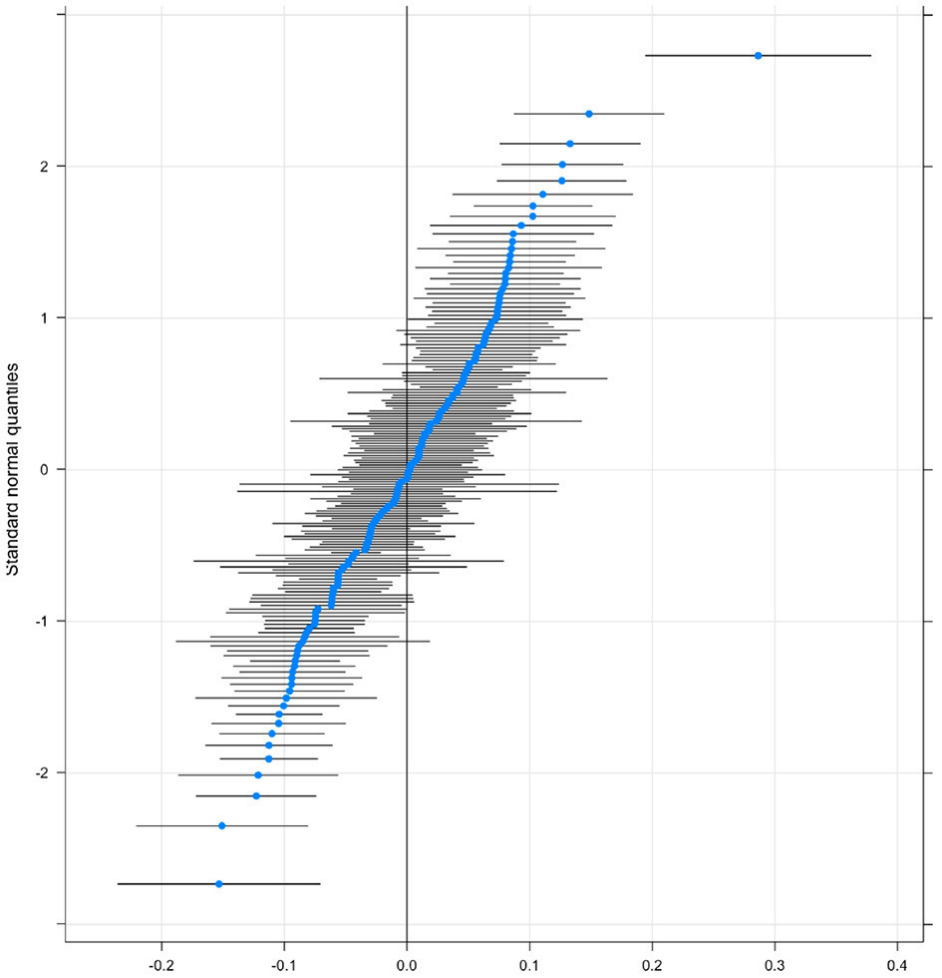
Random slope of income on low experiential SWB.

In our estimations up to now we assume that the relationship between individual factors and low SWB is the same across all countries. In other words, individual- and country-level features do not interact. There are good reasons to think that this may not hold. For example, the role of higher income in protecting people from low SWB might be expected to vary across places, even when controlling for the overall economic prosperity of a country, for example, due to differences in welfare provision. This is what we turn to next. To test that we run a random slope model that allows for cross-country heterogeneity in the relationship between income and both types of low SWB. Figure 4 plots the results showing that there are a lot of countries on the top right (and bottom left) of the income graph where the effect of income is significantly more (less) important for low SWB. A list of each country where income has a significantly above or below-average correlation with income is included in Supplementary Tables 6, 7 for low evaluative and experiential wellbeing, respectively. Of the 164 countries in our sample, a fifth of them has a significantly higher than the average relationship between income and low evaluative wellbeing and just over 18% have a below-average one. Similarly Figure 5 plots the same relationship for low experiential well-being.

## 4. Discussion

SWB research has generated important insights into the predictors of people's evaluations of their lives and their feelings as they go about them. However, to date, this research has typically focused on wellbeing at the mean (for exceptions, see Binder and Coad, 2011; Dolan et al., 2022), and most often looked at individual level factors within a given country. In the current work, we examine the predictors of low SWB, both in terms of low life evaluations and negative affect on the previous day, and go beyond an individual country to look across 164 countries and five world regions using the GWP. In doing so, we provide insights into the relative importance of individual, time and country-level factors in determining people's likelihood of reporting low SWB.

The results yield a number of important insights. First, across both measures of low SWB, who you are matters far more than the country you live in and, to an even greater extent, the year you report your wellbeing. These individual factors capture everything about a person that is not attributable to the country they live in, including sociodemographics like gender, age, education etc. but also their genetic makeup and time use (both important determinants of wellbeing identified in previous literature (Rietveld et al., 2013; Brand et al., 2020), as well as many unobservable characteristics. These results speak to the question posed at the beginning: behind a veil of ignorance what you would want to know to better understand your risk of reporting low SWB? The answer across both measures is clearly who you are.

Second, when comparing the relative importance of individual, country, and time level factors across both measures of low wellbeing, we see that country-level predictors explain substantially more of the variation in low evaluative wellbeing than they do low experiential wellbeing. In other words, the country you are living in is more predictive of the probability of evaluating your life poorly than it is of feeling a range of negative emotions for most of the day yesterday. When people are asked to place themselves on a rung of ladder that runs from the worst possible life to the best, this requires people to have some concept of how both of these types of lives look. In order to rate themselves as having the worst possible life or close to that (the bottom four rungs in our definition of evaluative SWB), people must have better lives in mind. Our results suggest that country-level factors play a more important role in that comparative process than they do in reporting on negative emotions, particularly for those living in Europe and North America.

When we include the individual level factors: income, gender, age, health issues, being able to count on help being retired, married, and living in an urban area we find these variables to be statistically significant and to predict low SWB in the same directions previously identified in the existing literature (with the exception of urban which is negatively associated with reporting low SWB). At the same time, however, the variance decomposition remains largely unchanged indicating that other unobserved individual characteristics are responsible for much of the variation in the likelihood of reporting low SWB across both measures. In contrast, the variance in low evaluations explained by country-level factors is substantially reduced (from 25% to 8%) once GDP per capita is included. The variance in low experiential wellbeing explained remains largely stable. These results suggest that a substantial part of the role of country-level factors in low life evaluations, but not low experiential wellbeing, is attributable to differences in economic conditions across countries. Importantly though, even when controlling for GDP per capita, country-level variation remains across both measures. Previous work by Diener and Tay (2015) using the GWP suggests environmental health, equality and freedom in nations likely all play an additional role, while work by Heukamp and Arino (2011) suggests religion, culture, and corruption may also contribute.

Third, individual, country, and time factors do not exist in isolation. These factors represent a complex system in which who a person is, interacts with their country-level environment, and time trends to determine their risk of low SWB. To evidence this, in the current work, we look at one individual-level determinant of SWB, which has been the focus of much academic attention—income (Kahneman and Deaton, 2010; Killingsworth, 2021). More specifically, we examine the importance of income as an individual-level predictor of low SWB, allowing it to vary across all of the countries in our sample. Our results indicate that how much money you earn has significantly different associations with your chance of reporting low evaluative well-being and low experiential wellbeing depending on your country of residence. Social capital, inequality, welfare provision, and other institutional factors likely play a role in explaining this finding (Helliwell and Putnam, 2004; Mikucka et al., 2017). For example, existing work indicates that income and inequality levels interact to predict wellbeing (Macchia et al., 2020). The most important takeaway for our purposes, however, is that the importance of income varies depending on the country in which you reside. This finding echoes other work on smaller samples of countries (Stanca, 2010). Though we do not examine this in the current work, research suggests the importance of income and other individual factors will likely also vary across time (Arrondo et al., 2021).

The social indicators movement assesses both objective welfare measures and subjective measures of psychological satisfaction and wellbeing in order to examine and track the quality of life (Land, 1983). While work on objective welfare measures has often looked at negative outcomes, for example in the multidimensional poverty index and the gender inequality index (Land and Michalos, 2018) far less of the work on subjective wellbeing has considered the bottom of the wellbeing distribution. Furthermore, while we have some existing evidence to suggest that the relationships between objective factors and subjective wellbeing will vary across countries with different social, economic, political, and environmental conditions (Macchia et al., 2019) as is the case in the social indicators literature more broadly (Land and Michalos, 2018) little multilevel analysis of these relationships exist. Our work addresses these gaps by considering the variance in low subjective wellbeing across individuals, countries, and time.

We are aware that our approach is not without its limitations. In terms of identifying which individuals have low evaluative and experiential SWB, we make a number of technical assumptions about how these conditions would be reported through the World Poll. That our evaluative SWB measure refers to life overall and the experiential measure relates to feelings yesterday is reflective of these two levels of wellbeing being by definition on different time scales. It is important to highlight, however, that we assume that reports of feelings yesterday act as an adequate proxy measure for experienced well-being (as is assumed in the SWB literature more broadly). That assumption should be kept in mind in interpreting the different results across the two measures. We also adopt arbitrary thresholds to identify low evaluative and experiential SWB. We examine the implications of these thresholds by using less strict criteria and results do not materially change (see Supplementary Tables 5, 6). Another assumption is that our measures of well-being are valid. The Cantril Ladder asks people to compare their life with an ideal and preferences is comparative in nature. Thus this item is seemingly more consistent with the preference satisfaction account of welfare rather than the mental state account that aligns with subjective wellbeing (Angner, 2010; Hausman, 2011).

A further limitation, that pertains to all cross-country SWB research, is the extent to which the well-being constructs and SWB measures translate across cultures. Gallup carries out extensive testing of all the measures included in the GWP and only includes those that they consider working across all of the counties in their sample. This robust approach (Gallup, 2021) provides reassurance that cross-country differences in low SWB that we identify are real differences in wellbeing as opposed to artifacts arising from different understandings of the questions being asked. The consistency of the results across different definitions of low SWB (see Supplementary Table 5) provides further reassurance.

We also cannot make causal claims based on our analysis. Like other correlational SWB research, the associations we present are vulnerable to reverse causality and omitted variable bias. As a result, insights from the current work do not suggest how to address people's low SWB but rather identify what parts of its variation can be explained by individual characteristics and time and country-level factors. We do not investigate mechanisms behind the patterns we identify, crucially, human attention (Dolan et al., 2021). Attention underlies one of the most prevalent lessons from SWB research—adaptation—and explains the consistent finding that income has diminishing marginal returns on SWB (Di Tella et al., 2010). More income matters most to those with the least of it because they focus attention on scarce resources, whereas more income is less noticeable to the wealthy (Layard et al., 2008). Our analysis emphasizes the importance of considering how and why individual and country-level factors may interplay to make people more or less vulnerable to low SWB.

It is perhaps not surprising that individual-level predictors of SWB are most closely associated with individually reported and analyzed SWB. An alternative approach to analysis is to consider SWB at the level of communities, which may be average SWB at the country level, or at lower geographical levels such as states and local authorities, or workplaces and schools (Deaton and Stone, 2013). It would then be possible to ask questions such as how do different features of communities shape community-level wellbeing and what can this tell us about how to intervene at local levels? What are the risk and protective factors for the low SWB of countries over time that national and global policies could intervene to shape? Such an approach moves us away from an individual focus to looking at social networks and considering wellbeing as a property of places and people, which public health interventions that draw on systems and place-based assets can shape (Krekel et al., 2020; Atkinson, 2021).

Finally, we examine a limited set of factors at the individual level and only include GDP at the country level. There are many more determinants of SWB, both individual and country level, identified in the existing SWB literature. While the factors we include represent many of the key determinants, future work could include a wider set of variables including for example unemployment, inequality and governance, thereby providing insights into the extent of the remaining unexplained variation at all levels.

Notwithstanding these limitations, the current work makes significant contributions to our understanding of what predicts low SWB across the world. In step with trends in the social indicators movement more broadly, it asks what matters for wellbeing, not on average, but for the worst-off, and how that varies across countries. The analysis we present demonstrates the value of going beyond an individual focus, to examine the complex interplay between people, places, and time in order to uncover who is at risk of falling among the world's worst off.

## Data availability statement

The data analyzed in this study is subject to the following licenses/restrictions: Access under individual license with Gallup. Requests to access these datasets should be directed to GM, g.melios@lse.ac.uk.

## Author contributions

GM: conception, design, data analysis, acquisition, and writing and editing draft. PD: conception and design, data acquisition, and editing draft. LK: conception and design and editing draft. KL: conception, design, data analysis, and writing and editing draft. All authors contributed to the article and approved the submitted version.

## Footnotes

^1^See Gandelman and Hernández-Murillo (2009) and Diener et al. (2018a) for work that goes beyond looking at average SWB and, speaking to those below neutral and average, respectively.

^2^Details on the questions asked and sampling are available through the Gallup World Poll Methodology (Gallup, 2022). Some of the results include fewer countries since not all nations were surveyed on the variables of interest throughout the survey. Further information on the GWP, sampling methodology, and coverage are available in the SM.

^3^For income we use a measure that uses Per Capita Annual Income in International Dollars to divide respondents into five groups of equal size. This provides a measure of respondent wealth that is relative to other respondents in that country.

^4^With the exceptions of South Africa, Botswana, Namibia, and Senegal.

^5^Data were collected by the World Bank Governance Indicators and refer to constant 2015 USD per capita GDP (Kaufmann et al., 2011).
