# Supplementary material for: Les Misérables: An analysis of low SWB across the world
Source: Front Psychol. 2023 Jun 8;14:1107939. doi: 10.3389/fpsyg.2023.1107939 (PMC10286796; doi:10.3389/fpsyg.2023.1107939)
Supplement: Supplementary file 1 [file Data_Sheet_1.pdf]

SUPPLEMENTARY MATERIAL

Gallup World Poll and Sampling

The analysis of this paper is conducted using the Gallup World Poll. Since 2005, Gallup has continuously surveyed residents in more than 160 countries and areas and in more than 140 languages, representing the views of more than 99% of the world's adult population. This makes it the largest representative survey of the world's adults. Unlike other surveys, the World Poll includes individuals who would not ordinarily be included in this type of research because of the difficulty and cost associated with reaching them. This includes individuals who can be surveyed only in person because they do not have access to a phone or the internet, people who cannot complete a survey by themselves because they are illiterate, people who live in rural and hard-to-reach areas, and residents of countries where the only available census data are out-of-date and infrequent.

In terms of sampling, with some exceptions, all samples are probability-based and nationally representative of the resident adult population. The coverage area is the entire country including rural areas, and the sampling frame represents the entire civilian, non-institutionalised, aged 15 and older population. Exceptions include areas where the safety of interviewing staff is threatened, scarcely populated islands in some countries, and areas that interviewers can reach only by foot, animal or small boat. Gallup uses telephone surveys in Northern America Western Europe, developed Asia, and Gulf Cooperation Council (GCC) countries. In Central and Eastern Europe, much of Latin America, former Soviet states, nearly all of Asia, the Middle East and Africa, an area frame design is used for face-to-face interviewing.

The typical Gallup World Poll survey includes interviews with at least 1,000 individuals. In some countries, Gallup over-samples in major cities or areas of special interest. Additionally, in some large countries, such as China and Russia, sample sizes include at least 2,000 adults. Although rare, in some instances, the sample size falls between 500 and 1,000.

Since in the World Poll, some of the surveys are conducted in person and some over the phone, one might be worried that the method of data collection might bias our estimates. In this section, we explore whether the main results presented in Table 2 differ by surveying method. About 500.000 surveys were conducted over the phone in 33 countries in our sample. Tables 5 & 6 show the results by subset. Results suggest that the surveying method does not systematically change the main inferences of our work.

	**Individuals**	**Country**	**Years**
Evaluative SWB			
Variance	3.29	0.53	0.02
ICC	85.50%	14.20%	0.30%
Experiential SWB			
Variance	3.29	0.21	0.02
ICC	92.57%	6.50%	0.93%

Table 5. Understanding Variance decomposition by level (in person interviews)

	Individuals	Country	Years
	Evaluative SWB		
Variance	3.29	1.03	0.01
ICC	75.92%	23.87%	0.21%
	Experiential SWB		
Variance	3.30	0.31	0.01
ICC	90.47%	8.56%	0.97%

Table 6. Understanding Variance decomposition by level (via telephone interviews)

Alternative definitions of low SWB

In the World Poll, Gallup identifies individuals as suffering (with low evaluative SWB) using a combination of 2 Cantril ladder questions: evaluation of life today and evaluation of life 5 years from now. If individuals respond to both questions between 0-4, they are considered to be suffering (low evaluative SWB). In this part of the supplementary material, we test whether ICC decomposition changes when using this alternative classification of low Evaluative SWB. Results remain virtually the same as described in the main body of the analysis. Moreover, we test whether lower levels of SWB (0-3 in the current Cantril Ladder) affect our inferences. In addition to that, we also check whether the presence of any 3 out 4 negative emotions (as an alternative definition of low experiential SWB) affects the variance decomposition in any significant way and results remain the same.

	Individuals	Country	Years
	Evaluative SWB		
Variance	3.29	1.19	0.01
ICC	73.26%	26,51%	0.23%
	Evaluative SWB (0-3)		
Variance	3.29	1.09	0.01
ICC	74.38%	25,52%	0.10%
	Experiential SWB		
Variance	3.29	0.46	0.01
ICC	87,80%	11,94%	0,26%

Table 7. Understanding Variance decomposition by level alternative Evaluative SWB measure

Evolution of measures over time

Figures 6 to 9 provide additional information regarding the evolution of low SWB across the sample period globally and split by global region. Figures 10 to 14 present the evolution of both measures on each country of the global regions. Figures show some striking examples of systematic differences between

evaluative and experiential SWB such as in Syria, Yemen or Benin as well as the significant well-being costs the recent crises had in Lebanon and Venezuela.

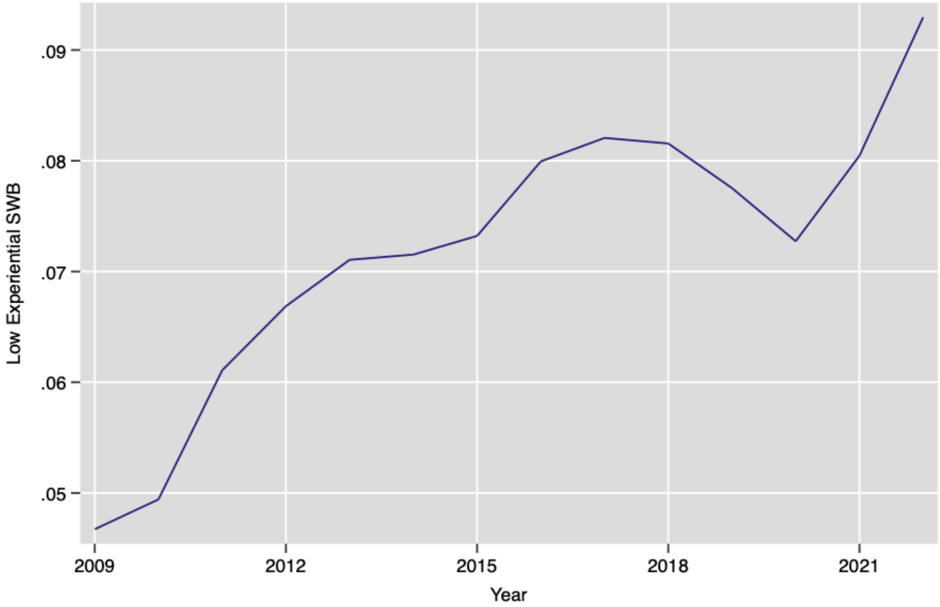

Figure 6. Evolution of low Experiential wellbeing around the globe

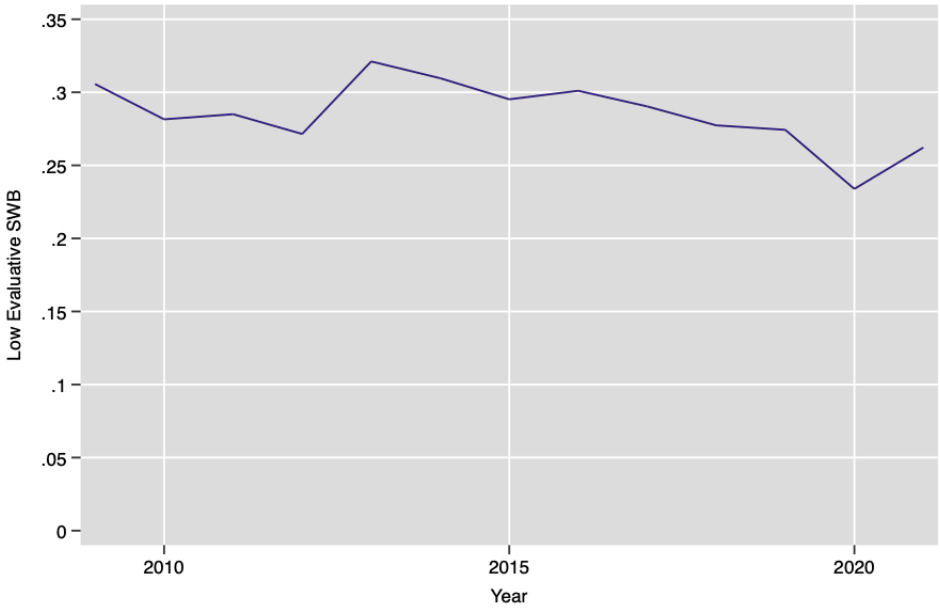

Figure 7. Evolution of mean Evaluative SWB around the globe

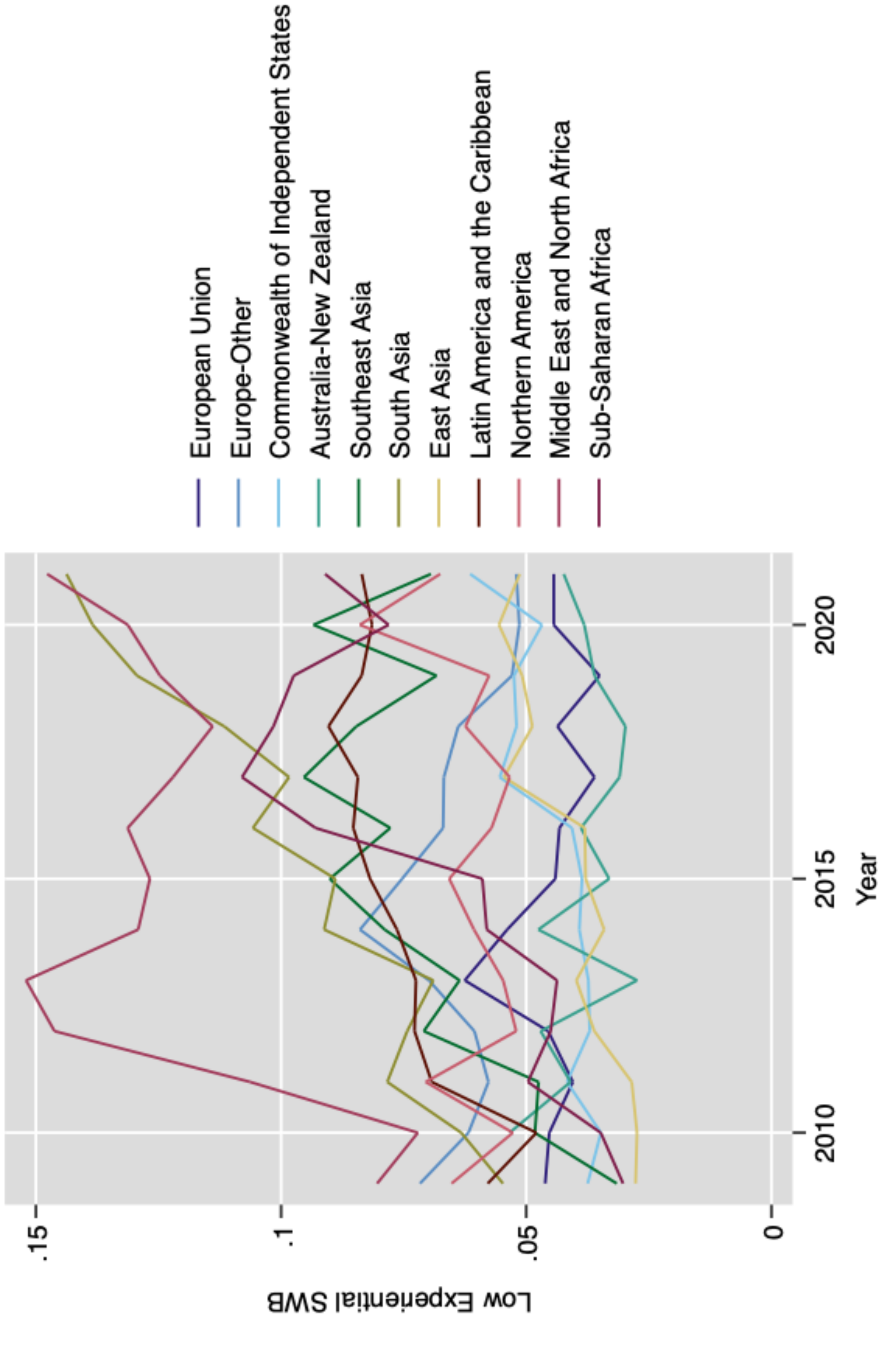

Figure 8. Low Evaluative SWB by Global region (average)

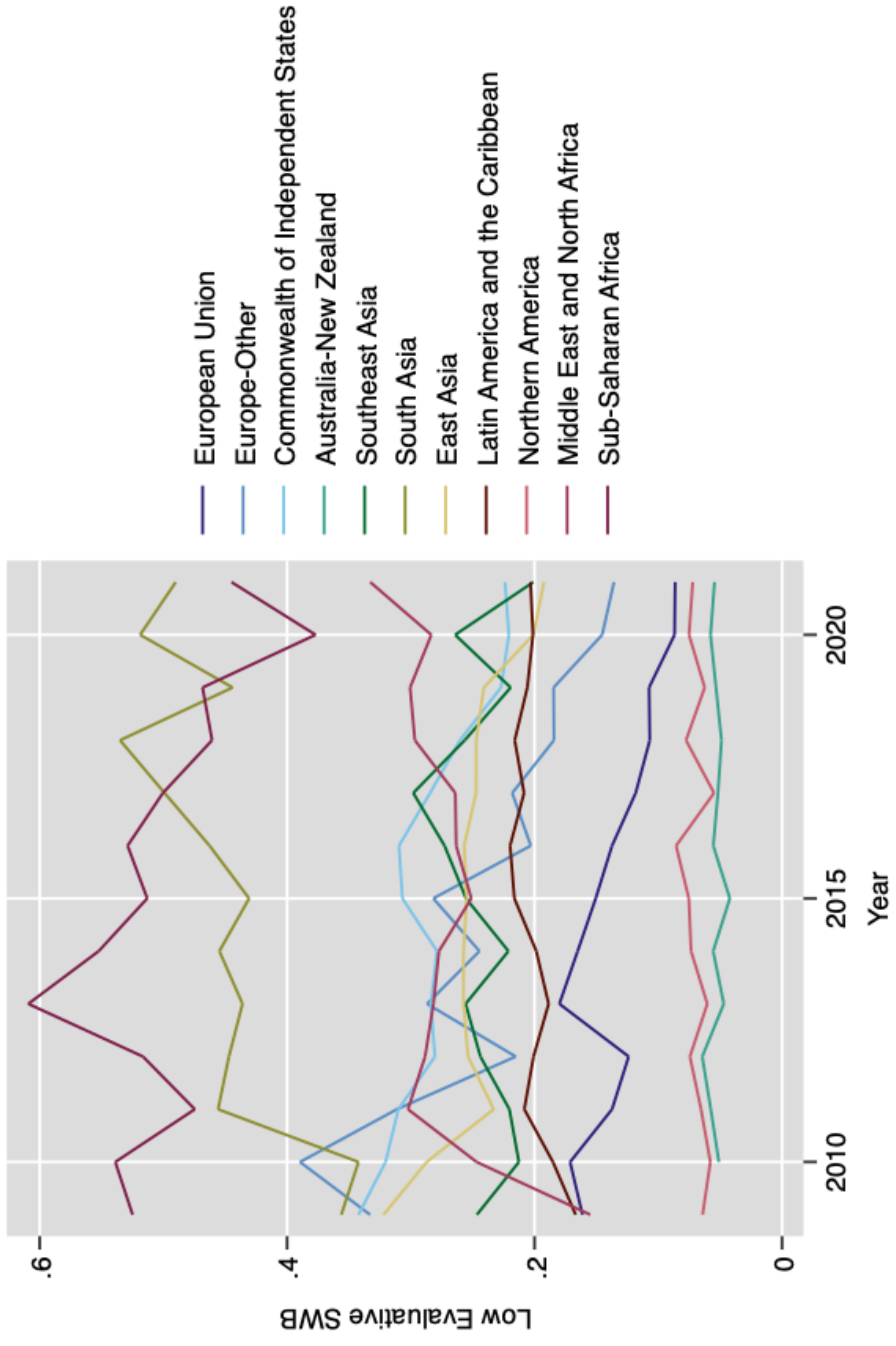

Figure 9. Low Experiential SWB by Global region (average)

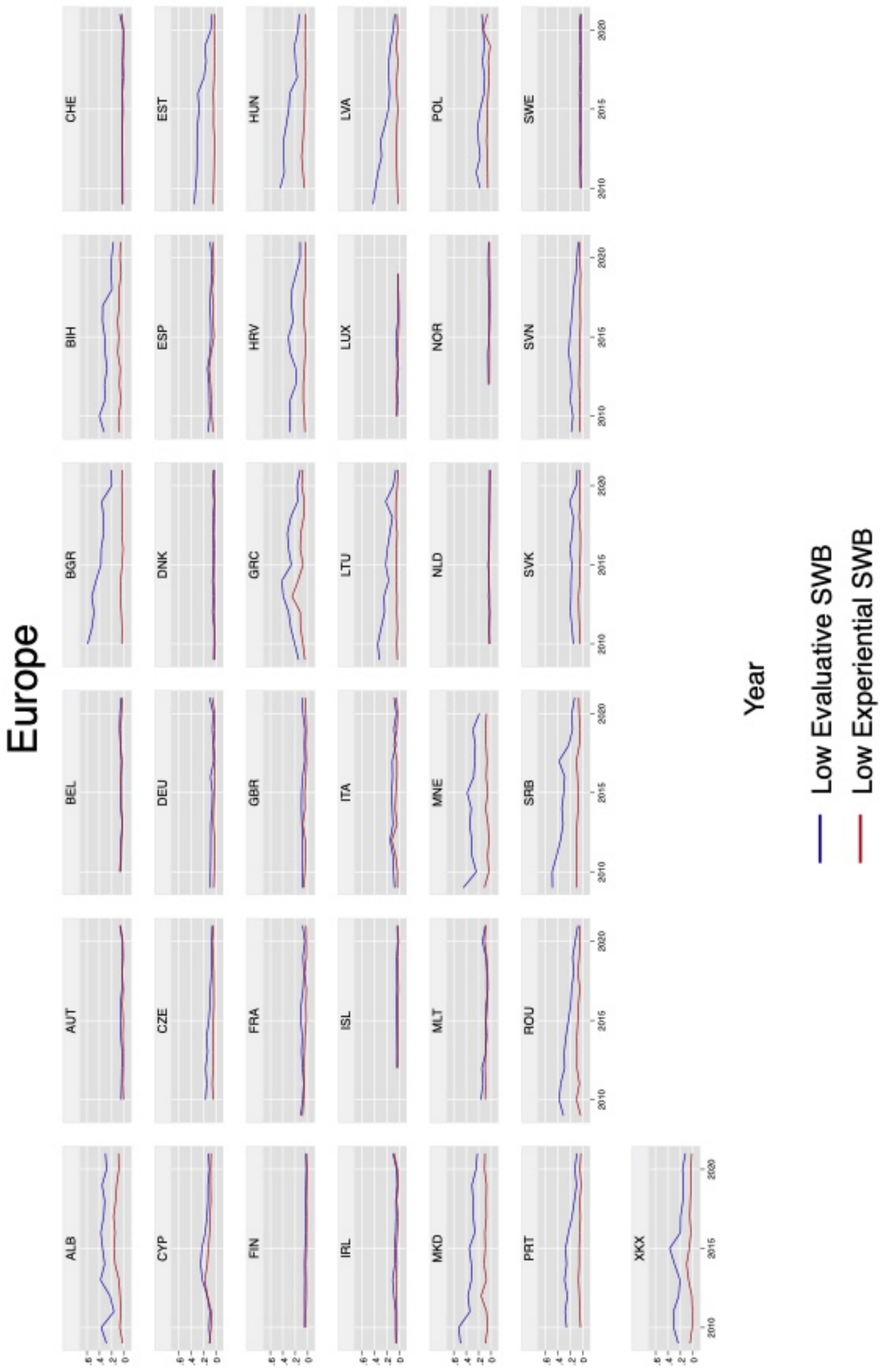

Figure 10. Evolution of low SWB in European Countries

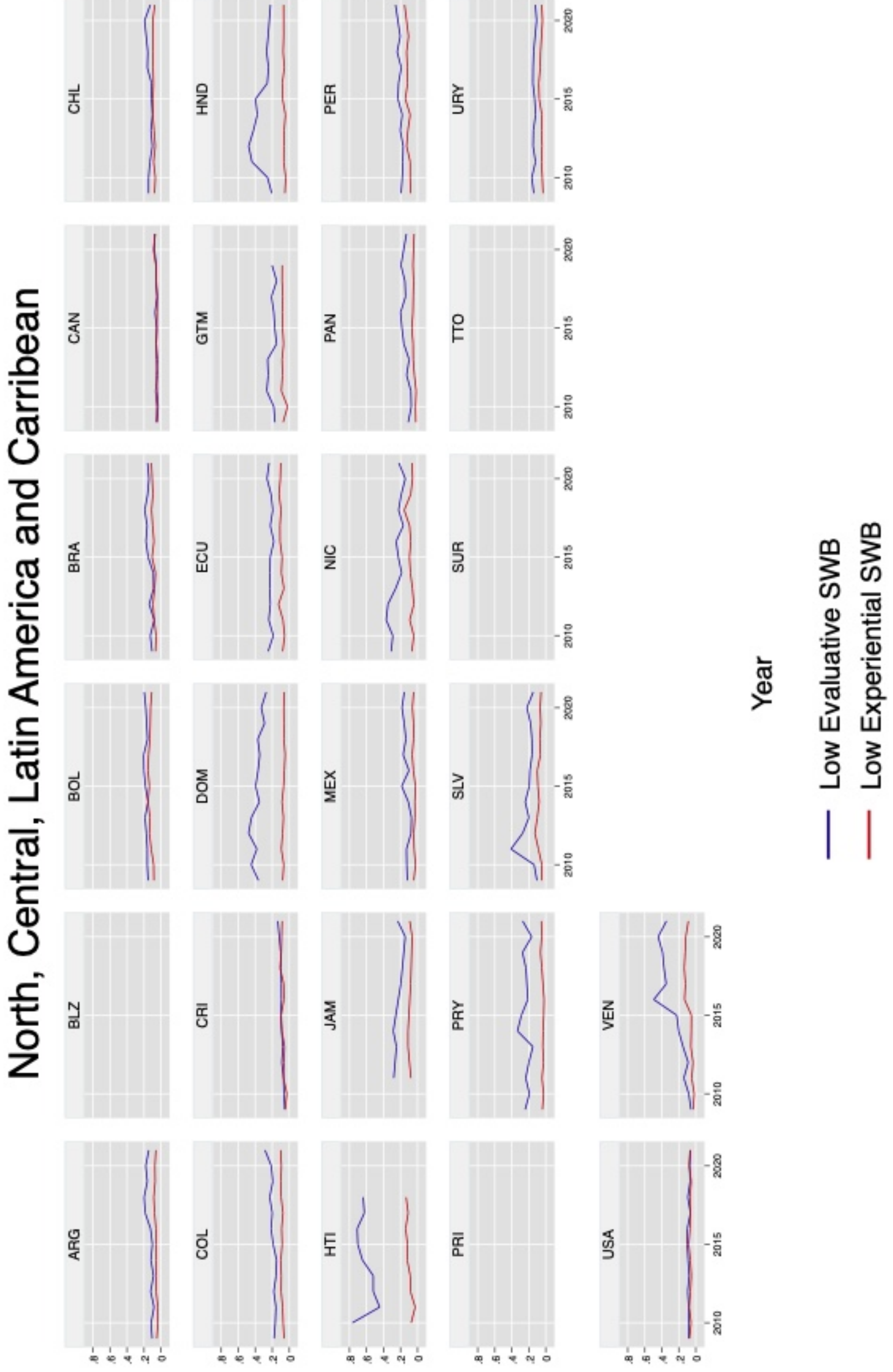

Figure 11. Evolution of low SWB in North, Central, Latin America & Carribean

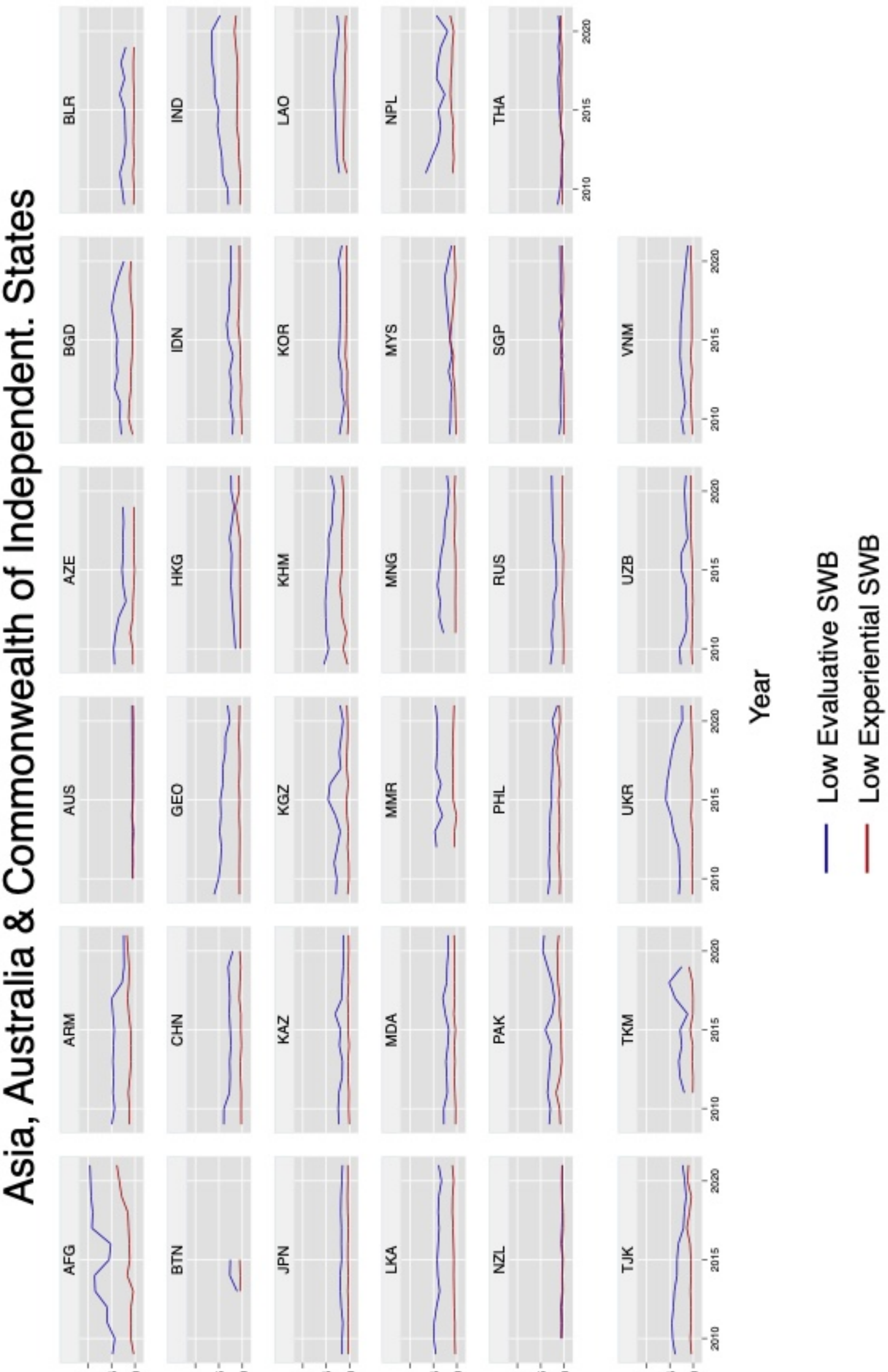

Figure 12. Evolution of low SWB in Asia, Australia, New Zealand and Commonwealth of Independent States

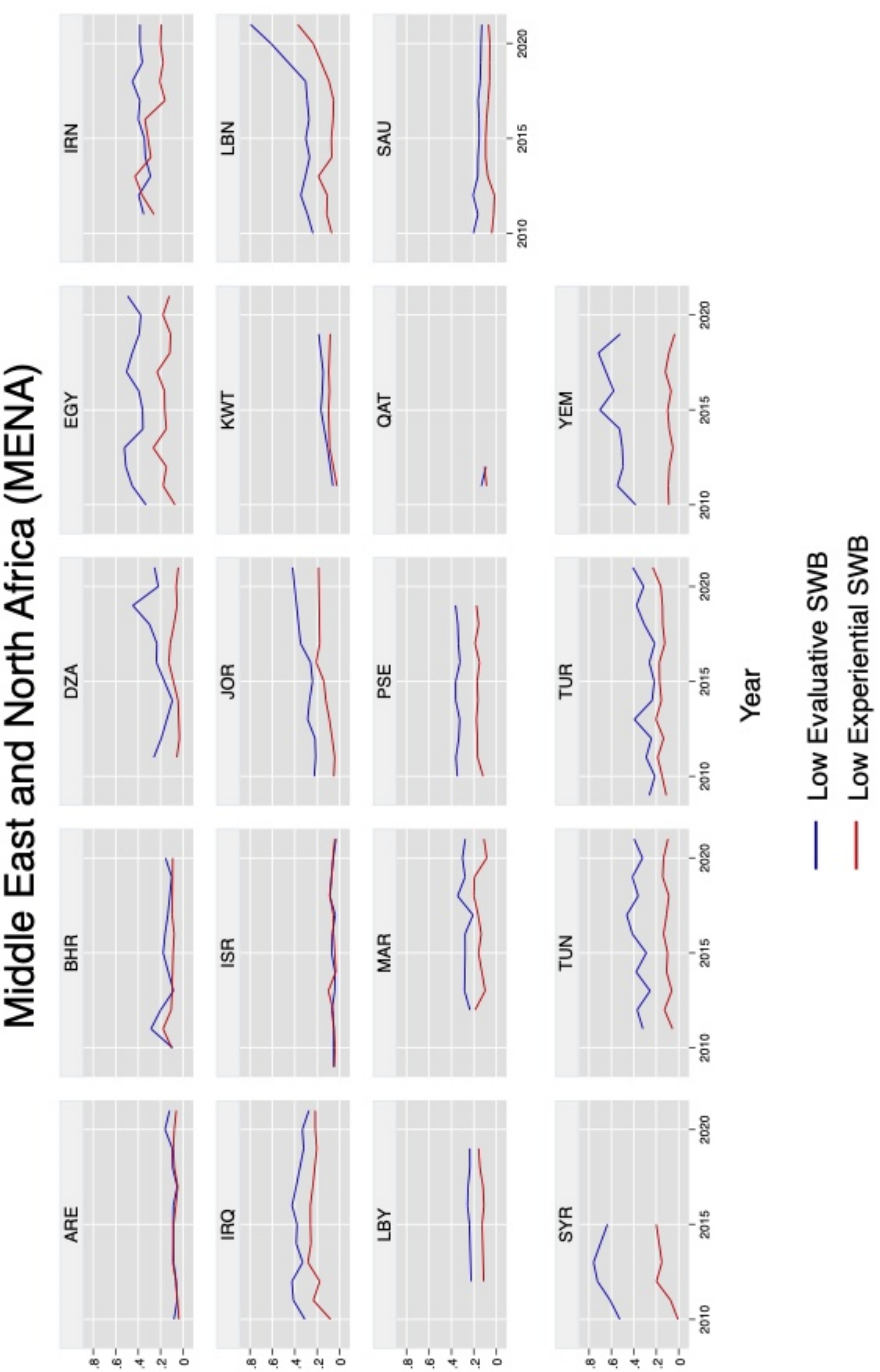

Figure 13. Evolution of low SWB in MENA Countries

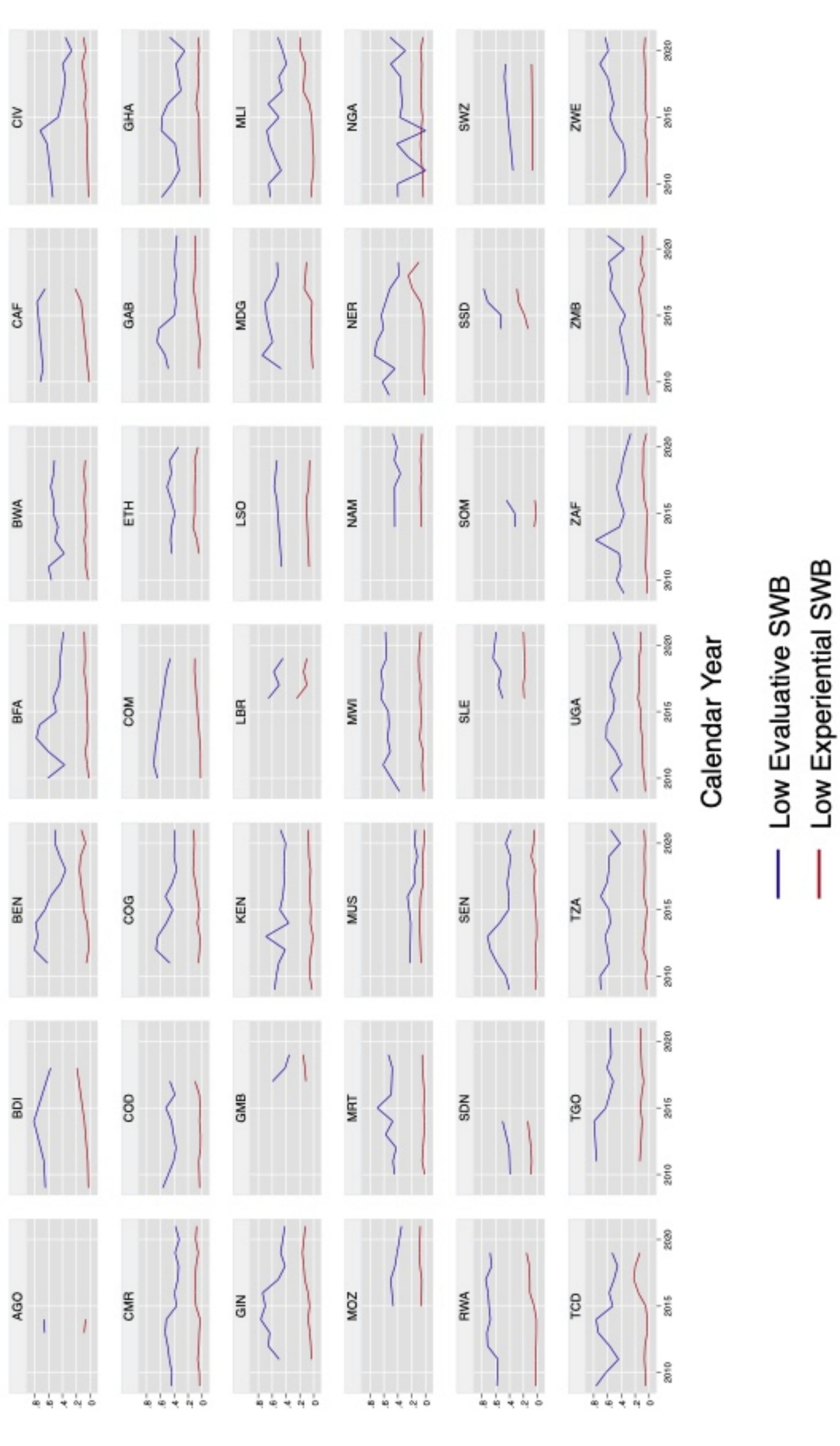

Figure 14. Evolution of low SWB in African Countries

Higher Effect of Income	Lower Effect of Income
Bahrain	Iran
Spain	Puerto Rico
Cyprus	Magadascar
Tajikistan	Lebanon
Bulgaria	Gambia
Botswana	Netherlands
Vietnam	Dominican Republic
China	Haiti
Sudan	Guinea
Tunisia	Benin
Austria	Niger
Ireland	Republic of the Congo
Australia	Nicaragua
Rwanda	Sierra Leone
Germany	Mauritania
Malaysia	Liberia
Palestine	Jamaica
Bangladesh	Armenia
Algeria	South Sudan
Finland	Venezuela
Democratic Republic of the Congo	Nigeria
Philippines	Belarus
Slovenia	Afghanistan
Iceland	Mali
Switzerland	Kazakstan
Indonesia	Uzbekistan
South Korea	Brazil
Montenegro	Turkmenistan
Zimbabwe	Kyrgyzstan
Angola	Syria
Singapore	
Peru	
India	

Table 8. Random Slopes of Income for Low Evaluative SWB

Higher Effect of Income	Lower Effect of Income
Norway	Dominican Republic
Netherlands	Bhutan
Lithuania	Turkey
Spain	Togo
Greece	Iran
Rwanda	Guatemala
Tunisia	Thailand
Germany	Botswana
Slovakia	Yemen
Romania	Central African Republic
China	Cambodia
Serbia	Namibia
USA	Benin
Australia	Burkina Faso
Bosnia and Herzegovina	El Salvador
Azerbaijan	Venezuela
Estonia	Nigeria
Chile	Jamaica
Ireland	Zimbabwe
Belgium	Burundi
Somalia	Honduras
Philippines	Mozambique
Brazil	Liberia
Palestine	Mongolia
Latvia	Senegal
Mauritius	South Sudan
Great Britain	Kyrgyzstan
Belarus	Nicaragua
Albania	Laos
Jordan	Kenya
Hungary	Côte d'Ivoire
Colombia	Guinea
Sweden	Qatar
Peru	Hong Kong
New Zealand	Myanmar
Argentina	Mali
Uruguay	Kazakhstan
FRANCE	Cameroon
Cyprus	Republic of the Congo
	Gambia
	Lesotho
	Sierra Leone
	Madagascar
	Syria
	Agola

Table 9. Random Slopes of Income for low Experiential SWB
